# More Similar than Different? Exploring Cultural Models of Depression among Latino Immigrants in Florida

**DOI:** 10.1155/2011/564396

**Published:** 2011-09-19

**Authors:** Dinorah (Dina) Martinez Tyson, Heide Castañeda, Milagro Porter, Marisel Quiroz, Iraida Carrion

**Affiliations:** ^1^Department of Aging & Mental Health Disparities, Louis de la Parte Florida Mental Health Institute, College of Behavioral and Community Sciences, University of South Florida, 13301 Bruce B. Downs Boulevord, MHC 1438, Tampa, FL 33612-3807, USA; ^2^Department of Anthropology, University of South Florida, 4202 E. Fowler Avenue, SOC 107, Tampa, FL 33620-8100, USA; ^3^Department of Mental Health Law and Policy, Florida Mental Health Institute, College of Behavioral and Community Sciences, University of South Florida, 13301 Bruce B. Downs Boulevard, Tampa, FL 33612-3807, USA; ^4^Rehabilitation and Mental Health Counseling, Florida Mental Health Institute, College of Behavioral and Community Sciences, University of South Florida, 13301 Bruce B. Downs Boulevard, Tampa, FL 33612-3807, USA; ^5^School of Social Work MGY 132, College of Behavioral Community Sciences, University of South Florida, 4202 E. Fowler Avenue, Tampa, FL 33620-8100, USA

## Abstract

The Surgeon General's report, “Culture, Race, and Ethnicity: A Supplement to Mental Health,” points to the need for subgroup specific mental health research that explores the cultural variation and heterogeneity of the Latino population. Guided by cognitive anthropological theories of culture, we utilized ethnographic interviewing techniques to explore cultural models of depression among foreign-born Mexican (*n* = 30), Cuban (*n* = 30), Columbian (*n* = 30), and island-born Puerto Ricans (*n* = 30), who represent the largest Latino groups in Florida. Results indicate that Colombian, Cuban, Mexican, and Puerto Rican immigrants showed strong intragroup consensus in their models of depression causality, symptoms, and treatment. We found more agreement than disagreement among all four groups regarding core descriptions of depression, which was largely unexpected but can potentially be explained by their common immigrant experiences. Findings expand our understanding about Latino subgroup similarities and differences in their conceptualization of depression and can be used to inform the adaptation of culturally relevant interventions in order to better serve Latino immigrant communities.

## 1. Introduction

In order to develop an evidence base for mental health care for minority populations, specific ethnic and cultural issues must be taken into account [1–4]. The US Surgeon General's report, “Culture, Race, and Ethnicity: A Supplement to Mental Health” and a recent white paper from the National Council of La Raza entitled “Critical Disparities in Latino Mental Health: Transforming Research into Action” [5, 6], points to the need for sub-group specific mental health research that explores the cultural variation and heterogeneity of the Latino population. Prior studies point to differences in depression and other psychiatric disorders among Latino ethnic subgroups [7–10]. In one of the largest epidemiological studies on Latino mental health, Alegria et al. report differences in the rates of disorders based on ethnic subgroup, age at immigration, and language proficiency [8]. The lifetime prevalence rate for major depression was reported to be 20.1% for Puerto Ricans, 18.6% for Cubans, 14.7% for Mexicans, and 13.9% for other Latinos. Not only are language issues recognized as a barrier to treatment, but cultural beliefs and practices influence the experience of depression (e.g., help-seeking behavior, symptoms, and ideas about etiology) and thus can moderate the effectiveness of prevention and treatment interventions [11]. One reason appears to be the conceptualization of depressive symptoms as social problems or emotional reactions to certain conditions, contrasted with the dominant perception of depression as a medical problem requiring medical treatment [9, 12, 13]. 

Overall, little is understood about the mental health experiences of Latino immigrants [14, 15]. Given the large number of Latino immigrants and the relevance of immigration status to service provision and to understanding the etiology of mental disorders, a focus on Latino immigrants is both timely and important [8, 16, 17]. Because of the heterogeneity of the Latino population, as the aforementioned reports suggest, there is a need for further research that disaggregates Latino immigrants to explore the nuances of differences in perceptions of depression and mental health services [11, 17, 18]. While there is a growing body of literature on Latino's perception of mental health [19–22], few studies have compared views of depression among the various ethnic groups that fall under the Hispanic umbrella, and even fewer have examined the role of immigration. While Latino immigrants appear to experience lower rates of depression than their U. S.-born compatriots and White Americans, they are also less likely to seek mental health services when they are depressed [6, 9, 20, 23–25]. Lackey (2008) suggests that if models used by immigrants to self-assess their mental health are different from the models used by clinicians, there will be a greater disparity between those who might need mental health care and those who are perceived to need it. Furthermore, treatment might be rejected if the treatment immigrants expect broadly differs from the treatment provided by clinicians [26]. A more nuanced understanding about the cultural construction of depression is needed to better serve Latino immigrant communities [20, 22]. 

This paper explores intracultural variations in descriptions of depression among Latino immigrants residing in West Central Florida. The first aim was to compare and contrast explanatory models of the causes, symptoms, and treatments for depression among four Latino immigrant subgroups. The second aim was to assess the level of agreement or disagreement between the different groups using cultural consensus analysis (CCA) as a first step to determine if there are shared cultural models of depression among Latino immigrants. The cognitive theory of culture [27, 28] postulates that culture is shared among members of the same culture and that members have a similar set of guidelines or model for a given domain, for example, depression [29, 30]. Thus, cultural models are cognitive schema that represent shared understandings of illness and recovery that construct meaning, represent social reality, and direct behaviors [31]. Previous research demonstrates a link between health behaviors and cultural models [32–34].

The population of Latino immigrants residing in West Central Florida saw a 57% increase from 2000 to 2010. This is a heterogeneous group with regards to nationality, immigration experience, and socioeconomic status. The four largest subgroups in the study area of Hillsborough County are Puerto Ricans (30%), Cubans (21%), Mexicans (21%), and Colombians (5%) [35]. The largest group, Puerto Ricans, have US citizenship status, which enables them to qualify for more services and facilitates travel between the mainland and their country of origin, allowing for the maintenance of family and social relationships. This is not the case for other groups, especially Cubans and Mexicans. While the Cuban population in this region has strong historical roots dating back to the 1900s, due to travel restrictions and the US embargo, they do not have easy access nor enduring ties to their homeland. The Mexican population is relatively younger, both in median age and in terms of historical immigration waves, and most reside in the rural parts of the county and are employed as agricultural workers. This isolation is compounded by long-term separation from family in Mexico due to increasingly strict border control and immigration regulations, and most encounter barriers to accessing services because of their immigration status. Finally, over the last decade, there has been a large influx of Colombians to the area seeking political asylum. Many are professionals with higher education although they are often relegated to work in the local service industry. Most retain strong social ties to family in Colombia and maintain frequent communication.

## 2. Materials and Methods

The study methodology consisted of ethnographic interviews that included the use of (1) structured (e.g., free-listed items) and (2) semistructured (e.g., open-ended items) data collection techniques [36]. The purpose of the interviews was to obtain culturally relevant descriptions of depression. The data presented in this paper reflect only information yielded during the structured (i.e., free listing portion) of the interviews. Free listing is a simple yet robust interviewing technique that is used to identify items in a cultural domain and to calculate each item's relative cultural salience (i.e., prominence, importance, familiarity, and representativeness) [36–38]. It requires three basic assumptions: items tend to be mentioned in order of familiarity, people who know more about the given domain list more than people who know less, and items most frequently listed indicate locally prominent terms [38, 39]. Thus, items listed earlier or more frequently are assumed to be more salient in a given domain [39]. Free listing is an effective method for defining the contents and boundaries of a cultural domain using the language, concepts, and categories that are meaningful to participants [37, 40]. 

### 2.1. Recruitment and Sampling Techniques

Purposive sampling techniques [36] including snowball and quota sampling were used to recruit participants. Inclusionary criteria included Latinos who (1) self-identified as foreign-born Mexican, Cuban, Columbian, or island-born Puerto Rican, (2) immigrated to the US after the age of 16, (3) were 18 years of age or older at the time of the interview, and (4) spoke Spanish. We drew our sample from established relationships with Latino community organizations. The sample size determination followed the method proposed by Weller (2007), whose algorithm suggests that without prior knowledge of the amount of agreement about a given domain (e.g., depression) among Latino subgroups, an average level of cultural competence would require a minimum 17 participants per group [41].

### 2.2. Data Collection

Face-to-face interviews were conducted at the participant's home or at another place convenient to them. Institutional review board approval was obtained from the University of South Florida. All study participants were informed of the goals of the study, the voluntary nature of their participation, and that their information would be kept confidential. The interviews were digitally audio recorded with the consent of participants. Each participant was asked to verbally complete the following free-listing exercise: (1) list all the things that can cause depression, (2) list all the depression symptoms you know about, and (3) list all the ways depression can be treated. Responses were written verbatim in the order they were listed. Nonspecific prompting and a rereading of the free list to participants was used to elicit as complete of a list as possible [42].

### 2.3. Data Analysis

The free list data was coded and analyzed in Spanish. Some items were recoded to standardize concepts for consistency [39]. For example, the terms “cries,” “cries all the time,” “cries a lot,” and “sobbing” were re-coded under the broad concept of “crying.” For the quantitative analysis of the free-listed data, the frequency of each item and the order of occurrence were calculated using the Anthropac [43] software program. Anthropac ranked the items by the order they were listed. We used the nonparametric chi-square statistical test to asses if differences between the groups were statistically significant. For the qualitative analysis of free listed items, we identified thematic categories that emerged from the data. Native Spanish speaking members of the research team (DMT, MP, and MQ) then independently grouped the items into thematic categories based on their similarity and then met to discuss the categories and come to agreement. For example, we grouped the items “being far away from family,” “migrating to another country,” “not understanding the language,” and “culture change” under the thematic category “immigration-related causes of depression.” 

Cultural consensus analysis (CCA) is a mathematical measurement model derived from the cognitive theory of culture [41, 44] that identifies the degree of shared knowledge within a group [45] or the shared agreement about a given domain across participants between and within groups [46]. CCA is suitable for analyzing within and across group similarities and differences [38, 47]. As described by Ross and Medin [19], we used CCA as a first step to explore cultural sharing regarding the causes, symptoms, and treatments for depression from the open-ended free-listed responses. 

We used the free-list procedure in Anthropac [43] to generate an item-by-participant matrix [48]. For this study, agreement was assumed when two participants either free-listed the same item or when they did not mention an item [49]. Cells contained a 1 if the participant listed the item and a 0 if they did not. Including zeros in cells when participants do not mention an item potentially inflates participant-by-participant agreement patterns. However, since free listing is not an exhaustive test but rather a sampling of salient items in response to a prompt, it is reasonable to assume that two participants who do not mention an item agree that the item is not salient at least in this context [48, 49]. In an effort to address the issue of potentially inflated participant-by-participant agreement patterns, we retained the free-listed items mentioned by at least 10% of participants in each ethnic group in the CCA. Thus, the sample size was slightly reduced for some of the ethnic groups in the CCA. For example, if a participant did not list the items mentioned by at least 10% of participants in each ethnic group for a given domain (e.g., symptoms of depression), they were excluded from the CCA analysis.

CCA creates a participant-by-participant correlation matrix (indicating agreement) among participants [50]. It is essentially a factor analysis of people, where the participants are the variables [41]. Consensus is indicated when there is a single-factor solution, which is when (1) the ratio between the first and second eigenvalues is high (usually a three to one), with a higher ratio indicating a stronger amount of agreement within the group; (2) all scores on the first factor are positive; (3) the first factor accounts for most of the variance [47]. In CCA, competence scores are also calculated for each participant in order to weight the response of each participant. Positive competency scores (between 0 and 1) are also a minimum requirement stating that there is a single factor solution, meaning a shared model [41, 46, 47, 51]. The competence score is not interpreted as the number of answers the individual knows, but rather how well the responses of the individual correspond with those of the group (e.g., ethnic group) [47]. 

First we ran the CCA for each ethnic group. Then, to test differences between the groups, we combined the four groups into a separate group called the “combined Latino immigrant group” and ran the CCA procedure again. Group differences can be identified in the following ways: (a) there is an overall consensus but it is greater within group than across groups and (b) not being able to achieve an overall consensus in light of the within group consensus [46, 50]. Thus, if the eigenvalues ratios were higher for the combined Latino immigrant group than for each individual Latino ethnic group, this would suggest a shared model among the Latino immigrants group in this study.

## 3. Results

### 3.1. The Sample: Participant Characteristics

Table 1 provides an overview of the 120 study participants, which consisted of 30 Colombian foreign-born, 30 Cuban foreign-born, 30 Mexican foreign-born, and 30 Puerto Rican island-born individuals. Women comprised the majority of the sample (66%). English proficiency varied, especially between Puerto Ricans and the other groups. A larger percentage of Puerto Ricans (60%) and Cubans (51%) had at least some college education. The majority of participants were married (53–70%). Colombians, Cubans, and Puerto Ricans had similar median ages (54, 57, and 52 years, resp.), while Mexicans had a much lower median age of 36. With regard to length of time in the USA, 59% of Cubans, 54% of Colombians, 43% of Mexicans, and 30% of Puerto Ricans had been in the USA ten years or less. A much higher proportion of Puerto Ricans (73%) had health insurance compared to the other groups. Experience with depression varied across groups, with 33% of Puerto Ricans, 30% of Cubans, 13% of Colombians, and 10% of Mexicans reporting that they had ever been diagnosed with depression.

### 3.2. Aim 1: Comparing and Contrasting Models of Depression Causality, Symptoms, and Treatments

**Figure 1:**
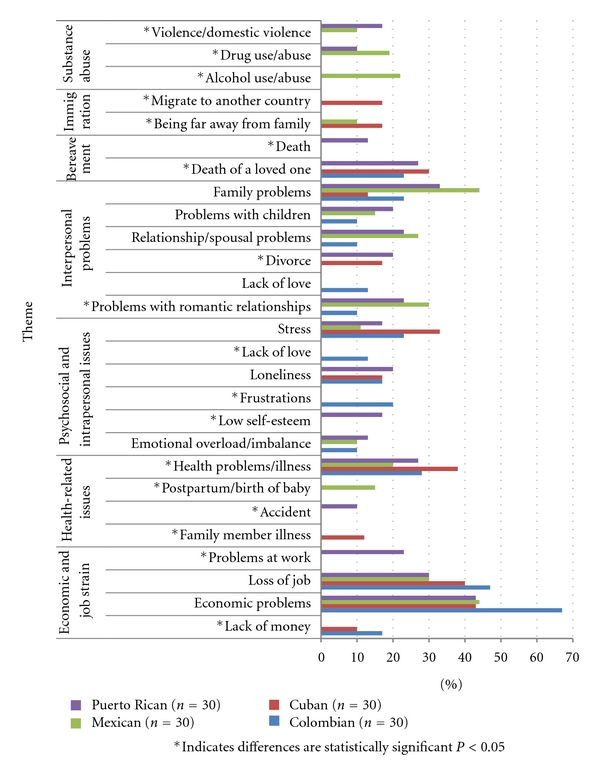
Most frequently listed causes of depression.

**Figure 2:**
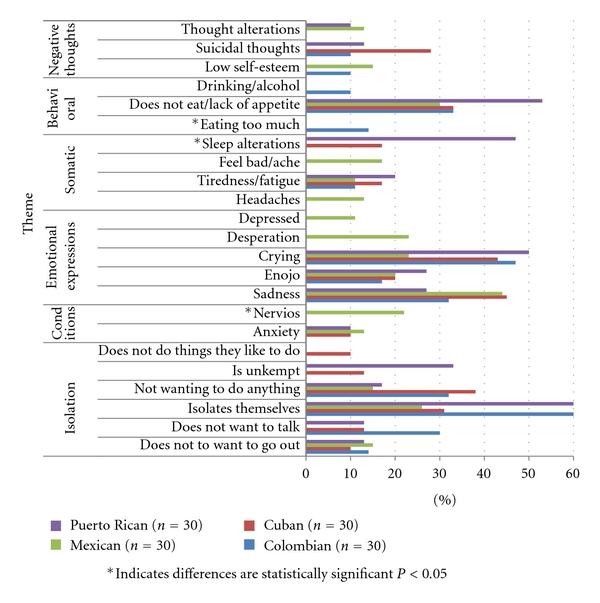
Most frequently listed symptoms of depression.

**Figure 3:**
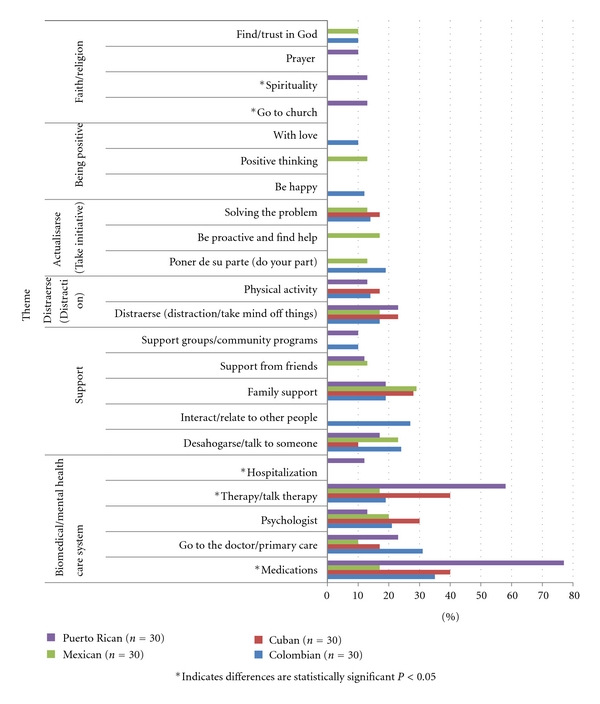
Most frequently listed treatments for depression.

Participants were asked to list the causes, symptoms, and treatments for depression. Below, the results of the most frequently listed items are first presented, including the thematic analysis of the free-listed data by domain. Figures 1–3 illustrate the most frequently listed items mentioned by at least 10% of participants in each ethnic group. Following this section, we present the agreement results from the CCA.

### 3.3. Causality

Figure 1 details the most frequently listed causal factors for depression grouped into the following overarching themes: (1) economic strain and work-related problems, (2) interpersonal problems related to family and or relationship issues, (3) physical illness/disease related, (4) psychosocial and emotional problems, (5) bereavement, (6) immigration related, and (7) substance abuse/violence. 

Work-related problems and economic strain that negatively affected the family such as financial insecurity, debt, unemployment, and lack of money were mentioned across the four ethnic groups. In fact, they were the most frequently listed causal factor for depression, with 67% of Colombians, 44% of Mexicans, 43% of Cubans, and 43% of Puerto Ricans mentioning this factor. For example, one Mexican participant said, “One falls in to depression in a situation like the one we are in today, because we don't have money to pay the bills.” Job loss or not finding employment was mentioned by all four groups. Health problems related to physical illness and disease were also mentioned as a possible cause of depression. Cuban (38%), Colombian (28%), Puerto Rican (27%), and Mexican (20%) participants listed the diagnosis of a terminal illness or living with a chronic disease as a cause of depression. Another health-related issue mentioned by 10% of Mexican participants was the birth of a new baby and associated postpartum isolation of the mother. 

 Psychosocial and intrapersonal issues were also mentioned as causal factors, with “stress” perceived as a cause of depression by 11–33% of participants. For example, a Colombian participant stated that it was “*la presión* (external stress or pressure) of life in the United States with the many obligations that one has, having to get to a certain place at this time, and so many responsibilities. Then, little by little, you start running out of time to do the things and then you get stressed and then you get depressed.”* La soledad* (to be alone) was also seen as a cause of depression rather than the other way around (i.e., depression causing loneliness or isolation), as illustrated by the following quote from a Cuban participant: “No one visits me, they don't call me, no one comes … and then I start to think about that and I start to cry and think bad things.” Another attributing factor listed was an emotional overload or imbalance as a result of “*un golpe fuerte emocional*” (a strong emotional blow/hurt or a situation that is too overwhelming). 

Interpersonal problems that negatively affected the integrity of family, such as conflict within the family, arguments, and discord, were mentioned by all four groups. This is illustrated by a quote from a Mexican participant: “… family problems can cause depression because they bring sadness … and then not getting along with the family.” Seeing their children in trouble or struggling was also mentioned. Many participants specifically mentioned problems with children who were “*descarrilados*” (“off track”), involved with drugs, disobedient, and/or unappreciative of the sacrifices that their parents had made for them. A Puerto Rican participant stated that depression can affect “persons that are confronting problems with their children … you know, the youth here get out of line and parents feel helpless to see their child, whom they love so much, doing drugs, lost, [and] that parent can get depression because of it.” Also mentioned were failed romantic relationships, divorce, and spousal problems such infidelity and unrequited love. 

Thoughts of death and the loss of a loved one were mentioned as causal factors for depression by Cubans, Colombians, and Puerto Ricans. Immigration-related issues, such as being far away from family and difficulties adjusting to life in the United States, were mentioned by 17% of Cuban and 10% of Mexican participants. A Cuban participant noted “I am not with my family a whole lot, I miss my mom very much, my sisters, and even though I am here with my son and my wife, I am sad because I am not with them. When my father died I was very sad because I could not be there.” Also mentioned were the sudden changes that immigrants face adjusting to the culture and life in the United States. 

Finally, depression was also attributed to substance use and domestic abuse. Twenty-two percent of Mexican participants specifically mentioned alcohol use. Trauma or violence due to physical or verbal abuse was also listed by Puerto Rican (17%) and Mexican participants (10%).

### 3.4. Symptoms

Figure 2 details the most frequently listed symptoms of depression grouped into the following thematic categories: (1) lack of interest and or anhedonia, (2) other conditions or mental illnesses, (3) emotional expressions, (4) somatic expressions, (5) behavioral, and (6) cognitive/thought-related symptoms. 

Symptoms related to lack of interest and anhedonia were mentioned by participants in the four groups. Becoming isolated was the most frequently listed symptom of depression among Colombians (60%) and Puerto Ricans (60%), while sadness was the most frequently listed symptom by Mexicans (44%) and Cubans (45%). As delineated in the following statement regarding symptoms of depression, one participant stated it was noticeable, “when a person becomes a hermit, they close themselves off, they don't want help, they cry a lot and nothing matters.” Also mentioned was not wanting to talk, go out, or not having “*ganas*” (desire) to do anything. *Enojo* (anger or rage) was also listed as a symptom of depression by participants in all four groups. 

Anxiety was identified as a symptom of depression by Mexicans (13%), Cubans (10%), and Puerto Ricans (10%). Interestingly, Mexican participants (22%) were the only ones to list *nervios*, a culturally significant and discrete syndrome or folk illness [30, 52], as a symptom of depression. Somatic symptoms of depression were also listed by participants across all four ethnic groups. However, there was variation in the specific symptoms reported. Tiredness and fatigue were mentioned by 11–20% of participants. Headaches and physical aches were only mentioned by Mexicans (13% and 17%, resp.). Sleep alterations such as sleeping too much and insomnia were also mentioned by 47% of Puerto Ricans and 17% of Cubans.

Behavioral symptoms related to loss of appetite were listed by 30–53% of participants. However, excessive eating was only listed by Colombians. Drinking alcohol as a symptom of depression was also only mentioned by 10% of Colombians. 

Negative thoughts such as suicidal ideation and feeling worthless were listed as symptoms of depression. Suicidal thoughts were mentioned by 28% of Cubans, 13% of Puerto Ricans, and 10% of Colombians but were not listed by Mexican participants as a symptom of depression. Thought alterations such as thinking too much, being too pensive and not being able to concentrate were also listed as symptoms of depression by Mexican (13%) and Puerto Rican (10%) participants.

### 3.5. Treatment

The treatment items listed can be grouped into the following thematic categories: (1) biomedical and or mental health care system, (2) support, (3) *distraerse* (distraction/doing things to take your mind off things), (4) *actualizarse* (take personal initiative), (5) being positive, and (6) faith/religion. 

Participants in the four ethnic groups listed medications, going to the doctor (i.e., primary care physician), therapy, and going to a psychologist. Interestingly, a much higher percentage of Puerto Ricans listed medication as a form of treatment (77%) compared to the Mexican participants (17%). 

Participants in all four ethnic groups listed support from family (19–29%) and being able to *desahogarse *(experience relief by talking to someone) (10–24%). *Distraerse* (distraction or activities to take one's mind off things) was listed by participants in the four ethnic groups (17–23%). One Puerto Rican participant said she, “thought it was important to find an activity, something the person likes to do, like gardening, walking.” Physical activity or staying active was mentioned by 17% of Cubans, 14% of Colombians, and 13% of Puerto Ricans but was not mentioned by the Mexican participants. 

*Actualisarse *(taking personal initiative) and solving the root problem was listed by Cuban (17%), Colombian (14%) and Mexican (13%) participants. The following quote from a Cuban participant illustrates this point: “Resolving the problems that are at the source and finding solutions for them is the first place to start.” 

Ten percent of Mexicans listed positive thinking. Being positive (10%) and happy (12%) was mentioned by Colombians. For example, a Colombian participant exuberantly said, “Ohhh, I think that it is all in your mind, if the person focuses on the positive side of things and they go through a bad experience, and they focus on the good things that they can gain from that experience then they won't go into depression, that is what I think.” 

Items related to finding something spiritual, going to church, and prayer were mostly listed by Puerto Rican participants (10–13%). To “trust and look/find God” was listed by Colombians and Mexicans (10%). Cubans did not list faith and, or religion-related items as a treatment for depression. 

### 3.6. Aim 2: Exploring Levels of Agreement/Cultural Sharing among the Four Latino Groups

Despite the range of responses and different amounts of items listed by each group, as described above, we also explored the levels of cultural model agreement within and between all four groups. In the following section, we explore the amount of agreement between Colombian, Cuban, Mexican, and Puerto Rican participants regarding the causes, symptoms, and treatments for depression.

### 3.7. Agreement on Causes of Depression

The eigenvalue ratios for causality were above the 3 : 1 ratio, suggesting agreement within each ethnic individual group. For Colombians, the eigenvalue ratio was 3.5 with an average competence of  .60 (s.d. =  .16), for Cubans (3.7 ratio) average competence of  .62 (s.d. =  .18), Mexicans (3.4 ratio) average competence  .60 (s.d. =  .17), and Puerto Ricans (3.6 ratio) average competency  .58 (s.d. =  .18). 

However, when the four Latino immigrant groups were combined, the eigenvalue ratio was considerably higher at 16.13 with an average competence of  .81 (s.d. =  .08), suggesting that there might be a shared model of depression causality. As a result, one preliminary conclusion based on this sample is that Colombians, Cubans, Mexicans and Puerto Ricans immigrants show strong intragroup consensus in their model of depression causality.

### 3.8. Agreement on Symptoms of Depression

The eigenvalue ratios for symptoms of depression were all above the 3 : 1 ratio. When the four Latino immigrant groups were combined, the eigenvalue was 8.3, or higher than the eigenvalue for each of the individual subgroups (5.1 with an average competence  .72 (s.d. =  .16) for Mexicans, 5.1 with an average competence of  .71 (s.d. =  .15) for Puerto Ricans, 3.6 with an average competence of  .63 (s.d. =  .24) for Cubans, and 6.4 with an average competence of  .74 (s.d. =  .15) for Colombians).

### 3.9. Agreement on Treatment of Depression

The eigenvalue ratio again was above the 3 : 1 ratio across all four Latino subgroups; for Colombians, it was 5.0 with an average competence of  .71 (s.d. =  .13), for Cubans 3.8 with an average competence of  .70 (s.d. =  .16), for Mexicans 5.6 with an average competence of  .70 (s.d. =  .16), and for Puerto Ricans 7.9 with an average competence of  .76 (s.d. =  .16). When all four groups were combined, the eigenvalue ratio was 11.5 with an average competence of  .83 (s.d. =  .07).

## 4. Discussion and Conclusion

This anthropologically informed study presents an innovative methodological approach to understanding depression by focusing on intracultural variations in perceptions of depression causality, symptoms, and treatments among Latinos immigrants. Despite a clear need to disaggregate ethnic subgroups rather than lumping all Latinos into one homogenous group without respect for historical, socioeconomic, and cultural differences, data from this study suggest a mostly shared model of depression among Latino immigrants in this region of Florida. Our results indicate more similarities than differences in how participants viewed the causes, symptoms, and potential treatments for depression. The high level of agreement across the three domains of depression causality, symptoms, and treatment with eigenvalue ratios of 3.0 or higher, and the lack of negative competence scores indicates a good fit to the consensus model. When comparing the subgroup eigenvalues to the eigenvalue of the combined Latino immigrant group across all three domains, the eigenvalue was considerably higher in the combined group. This suggests that there might be a shared understanding of depression among the Latino immigrant groups studied and is similar to other studies that have found a shared causal model of disease (e.g., breast cancer and *empacho, *a common folk illness) among Latino immigrants, regardless of country of origin [45, 53].

However, context and intergroup variation must still be taken into account, as there were significant differences in the items that participants from different countries of origin listed as important. For example, more than 40% of Columbians reported concerns relating to stress and job loss, which may be related to their immigration status and unique history in Florida, as noted at the start of this article. Economic problems and postpartum issues were reported as causes of depression for Mexicans, who are also the youngest group in the sample and most likely to be of childbearing age. Further, 90% of this group did not have health insurance, and the birth of a child directly impacts the family's overall income. Among Cubans, physical illness, economic problems, and loss of job were identified as primary causes of depression; these three factors are interrelated for this population, who are the oldest in the sample and of whom 43% did not have health insurance. Among Puerto Ricans, family and economic problems were identified as the most common causes of depression. Thus, the saliency of stress and loneliness varied among participants and was in part influenced by legal status and immigration-related factors, especially in the case of Cubans and Mexicans.

The majority of Latino immigrants across the four groups recognized depression in terms that would be utilized by mental health practitioners. This is similar to the findings from Martinez Pincay and Guarnaccia (2007), in which participants listed emotional and somatic symptoms of depression comparable to those in the DSM-IV diagnostic manual [21]. Consistent with the literature, we found that Latino immigrants' views of depression causality were primarily informed by social and interpersonal factors that attributed depression to life circumstances or interpersonal and economic problems, rather than to biochemical or biological factors [14, 19, 22, 26, 34, 54]. This appears to fit well with the “situational” model of depression, which underscores the relevance of contextual factors such as social problems, economic strain, and interpersonal conflicts [14, 19, 55–59]. In this approach, depression is not seen so much as an illness but rather a result of the hardships experienced and challenges faced living in the United States [21]. The prominence of interpersonal problems, family conflict, and economic problems highlights the importance Latino immigrants place on family unity and cohesion [19]. 

By identifying patients' cultural models of depression, clinicians and practitioners may be able to address the underlying sociocultural factors that impact diagnostic and treatment accuracy [33]. Our findings may have implications for the appeal of certain treatment approaches. For example, the salience of pharmaceutical treatments varied by group, with medications emphasized by participants in the Puerto Rican but not listed as an option for the Mexican group. However, this should not necessarily be viewed solely as a matter cultural preference, but rather related to access. In our sample, Puerto Ricans had the highest rates of health insurance and thus more access to medications, and Mexicans the lowest; in each case, access can explained by the groups unique immigration trajectory and related policies. 

On the other hand, *desahogarse *(gaining relief by talking to someone) and family support ranked highly for all groups, and these could represent important starting points for designing interventions. The saliency of interpersonal sources of support and the emphasis on problem solving might suggest that programs or interventions that build on social relationships and teach problem-solving and self-management skills to cope with situational problems might be well received by Latino immigrants, regardless of country of origin. Understanding that perceptions of depression are also informed by the immigration experience and adaptation to life in the United States can help practitioners assess, better engage, and treat patients from Latino immigrant communities. It may not be necessary to create programs for a specific Latino ethnic group (e.g., Cubans), but it is vital to create psychotherapeutic interventions and depression prevention programs that resonate with the saliency of social stressors and interpersonal relationships. 

This study has several limitations. First, we used a nonrandom purposive sample which limits the generalizability of results to Latino immigrants outside of West Central Florida or to US-born Latinos. The second limitation of this study is that it does not account for other social and demographic factors such as gender, education, income, and length of time in the US that may affect perceptions of depression causality, symptoms, and treatment. These will be addressed in a future study. Third, the use of the CCA technique with free-listed data is exploratory, with the verification of the sharing and distribution of cultural models requiring additional data [60] using a fixed-format questionnaire. Further studies are needed to confirm if there is indeed a shared model of depression among Latino immigrants and to compare the findings to non-Latino groups and clinicians' views of depression. 

Findings from this study contribute to our understanding about Latino immigrants' views about depression and its treatment by examining subgroup similarities and differences. Our preliminary results can inform additional studies on this topic and ultimately may aid in the adaptation of culturally relevant interventions to better serve Latino immigrant communities, regardless of country of origin.

## Figures and Tables

**Table 1:** Sample demographics: Latino immigrants.

	Colombian (*N*=30)%	Cuban (*N*=30)%	Puerto Rican (*N*=30)%	Mexican (*N*=30)%
English proficiency				
Very good	3.0	10.0	60.0	3.0
More or less	73.0	53.0	30.0	47.0
Not at all	24.0	37.0	10.0	50.0

Gender				
Women	67	67	67	67
Men	30	30	30	30

Level of education				
Elementary or less	7.0	13.0	10.0	30.0
Some high school	17.0	3.0	7.0	30.0
High school	13.0	33.0	23.0	37.0
Some college + plus	63.0	47.0	60.0	3.0
Vocational/technical	0.0	4.0	0.0	0.0

Marital status				
Single	30.0	3.0	20.0	13.0
Married	57.0	54.0	53.0	70.0
Divorced	13.0	30.0	23.0	17.0
Widow	0.0	13.0	4.0	0.0

Age				
Median age	54	57	52	36
Range	(24–77)	(23–86)	(26–77)	(18–62)

Length of time in us				
1 year or less	7.0	13.0	17.0	0.0
2–5 years	4.0	13.0	3.0	23.0
6–10 years	43.0	33.0	10.0	20.0
11 + years	46.0	41.0	70.0	57.0

Health insurance				
Yes	23.0	57.0	73.0	10.0

Ever been diagnosed with depression				
Yes	13.0	30.0	33.0	10.0
